# Saltwater Intrusion Function and Preliminary Application in the Yangtze River Estuary, China

**DOI:** 10.3390/ijerph16010118

**Published:** 2019-01-04

**Authors:** Zhi Xu, Jing Ma, Yajie Hu

**Affiliations:** 1 Department of Hyraulic Engineering, Tsinghua University, Beijing 100084, China; xuzhitc159@163.com; 2 China Institute of Water Resources and Hydropower Research, Beijing 100038, China; jingma@iwhr.com

**Keywords:** S-shaped curve, saltwater intrusion, MIKE 21, river discharge, tidal range

## Abstract

More attention has been paid to saltwater-intrusion-related problems in recent years. In this research study, a saltwater intrusion function in the Yangtze River Estuary (YRE) was constructed based on the theory of the interactions between energy accumulation and impedance. A MIKE21 model was used to simulate the hydrodynamics of the YRE. Then, through the analysis of the relationships between the river discharge conditions, tidal ranges, and saltwater intrusion, it was determined that, under certain river discharge conditions, the tidal ranges and salinity levels at the stations in the southern branch (SB) of the YRE conformed to S-shaped curve characteristics. Also, the tidal ranges and salinity excessive area rate (SEAR) displayed similar characteristics. Furthermore, the river discharge conditions were also found to match the S-curve characteristics between the two aforementioned relationship features. Therefore, the saltwater intrusion function of the YRE was constructed based on the previously mentioned development rules. Also, the applied quantification methods were elaborated, and the values of the parameters were determined. As a result, the critical river discharge (more than 10,000 m^3^/s) was obtained, which could withstand large-scale saltwater intrusions. When the river discharge was greater than 30,000 m^3^/s, the area was considered to be basically without salt water intrusions, and the estuarine ecology was in an optimal state. The saltwater intrusion losses from 2005 to 2015 are also calculated. These findings have important reference value for water dispatching of the YRE in the dry season.

## 1. Introduction

The estuarine area of the Yangtze River has become an area of rapid economic development and progress due to the abundance of freshwater and ecological resources and its advantageous geographical position. Meanwhile, as human activities are growing, the use of water resources continues to increase. Moreover, the amounts of discharge from many rivers have been observed to have decreased [1]. In particular, the duration of the low flows during the dry seasons has increased, with some rivers even drying up. As a result, the long-term balance of conventional salt intrusion has been damaged, and abnormal phenomena continue to increase [2]. It has been observed that there are increased diffusion regions, a higher probability of occurrence, longer durations, and earlier occurrence times. Therefore, the saltwater intrusions have become increasingly serious, with negative effects on the production and lives of the population in the area, and pose threats to the fragile ecological environment of the Yangtze River Estuary (YRE) [3]. In this study, the goal was to enhance the research regarding saltwater intrusion mechanisms and control factors in order to achieve results of theoretical significance and practical need [4]. In recent years, due to the construction of upstream river hydropower stations and reservoirs, as well as the rebuilding of the estuaries, increases in water levels along the river, and global climate change, the river runoff has displayed a definite decreasing trend, and salt water intrusions have increased [5]. Previous studies were mainly based on numerical simulation and influencing factors, and the research methods have included numerical simulation methods, theoretical analyses, and statistical models [6,7,8]. Also, mathematical modelling has been carried out using one-dimensional, two-dimensional, and three-dimensional models of the salinity spatial distribution, along with physical process simulations of the influencing factors [9,10,11]. Theoretical analyses have been used in the physical model testing methods, and experimental data have been used for the theoretical analyses in order to reveal the mechanism of the intrusions [12]. The statistical models were based on the combined effects of the runoff and tidal currents on the salinity in the estuaries, and multiple regression models for the salinity and runoff and tidal current changes were subsequently established [13,14].

In recent years, the frequency and intensity of saltwater intrusion in the YRE have continuously increased. Previous related studies have included numerical simulations of the changes in salinity in the YRE [15,16,17]. Hui et al. studied the impact of saltwater intrusion on water resources [18]. These have included the “Study of Seawater Intrusion and Utilization of Freshwater Resources in the YRE” [13] and the “Study of the Intrusion of Saline Water at Extremely Low Water Runoff Levels in the YRE during Extreme Drought Years” [19]. It has been established that the tidal and river flows are the most important factors affecting the YRE. Chen Jiyu put forward that the critical flow of saltwater intrusion seriously affecting freshwater resources is 1000 m^3^/s in the southern branch (SB) of the YRE [20]. Tong used mathematical models to reveal the potential salt water intrusion on the surface and bottom of the Qingcaosha Reservoir estuary [21]. The abovementioned studies often performed numerical simulations in order to analyze the processes of saltwater intrusions. Therefore, there continues to be a lack of research on the combination of numerical simulations and physical mechanisms of the saltwater intrusions in the YRE.

In this study, a saltwater intrusion function of the Yangtze River estuary was constructed based on the theory of the interactions between energy accumulation and impedance. The two main hydrological factors of the saltwater intrusion, tidal range and discharge, were then analyzed. The diffusion processes of the saltwater intrusions that were formed by the aforementioned two factors were derived. Then, the functional relationships of the diffusion of the saltwater intrusion were established, which confirmed the S-shaped curve of the diffusion process. It was determined that the river discharge during the dry season in the Yangtze River estuary is not less than 10,000 m^3^/s. The index has important reference value for water dispatching in the middle and upper reaches of the Yangtze River.

## 2. Materials and Methods

### 2.1. Study Site

**Figure 1:**
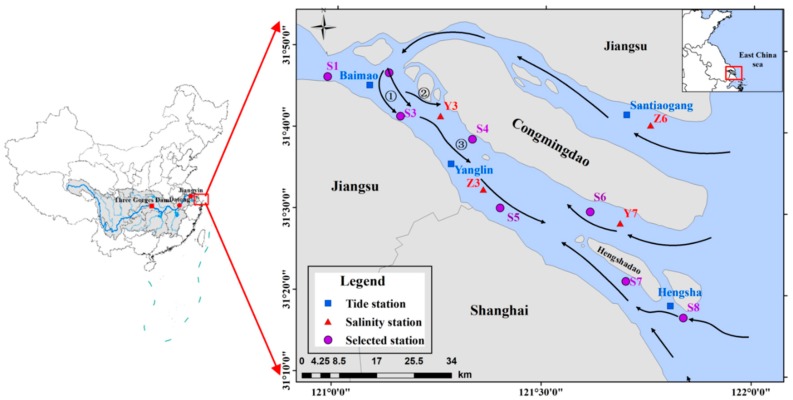
The Yangtze River Estuary.

The Yangtze River is the largest river in Asia. Under the influence of flow and current, it grows the river regime of the YRE [22], as shown in Figure 1. Located in the upper reaches of the Yangtze River, the Three Gorges Dam (TGD) is the largest dam in the world [23]. The impoundment of the Three Gorges Project caused the saltwater intrusion cycle in the YRE to advance, and the intrusion intensity increased. Due to the regulation and storage of the TGD, the river discharge increased in winter, and the saltwater intrusion was slowed to some extent.

Due to the influence of the YRE, the salinity of the northern branch (NB) has been high for a long time [24]. The tide in the NB may rise to Chongming Island and enter the SB [25]. Along with the decrease in the flow into the sea, the high-salinity saltwater of NB is poured into the SB (Figure 1) [26]. The lower reaches of the SB are affected by tidal currents, resulting in saltwater intrusion. In recent years, with the increasing water intake and related water conservation construction projects, the frequency and intensity of saltwater intrusion into the YRE have increased, which poses a threat to drinking water and causes economic losses [19,20].

### 2.2. General Description

#### 2.2.1. Hydrological Factors

**Figure 2:**
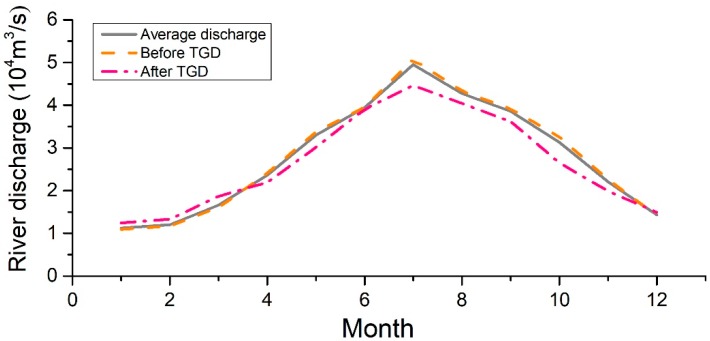
The average river discharge distribution of Datong station. TGD, Three Gorges Dam.

The average annual flow value of the YRE is 9.24 × 10^11^ m^3^ [16]. The average annual inflow rate is 27,856 m^3^/s, and the flow data from 1957 to 2015 of Datong Hydrological Station are adopted (Figure 2). The average runoff during the dry season is 16,542 m^3^/s.

The TGD is China’s largest hydropower project. It began to store water on 1 June 2003. Its construction has changed the distribution of the flow into the sea [27,28]. It can be seen from Figure 2 that, after the completion of the TGD, the flood season flow of the Datong Hydrological Station decreased slightly in the flood season, with an average discharge of 39,858 m^3^/s, and the average runoff in the dry season increased, with an average discharge of 16,867 m^3^/s.

The YRE is considered to be a meso-tidal estuary. It has a half-day tidal cycle, with two climaxes and low ebbs in a lunar day (approximately 24 h and 50 min) [29]. This study selected the Takahashi Station as a representative tidal station, and subtracted the highest tidal level and lowest tidal level adjacent to each other on a daily basis to obtain the two tidal range differences. The tidal range was taken as the tidal intensity of the current day. The daily tidal range of the year changed with time. Gaoqiao Station is located in the middle of the SB in the YRE. The data regarding the tide levels was determined to be complete, and objectively reflected the changes in the tidal levels in the SB of the YRE.

**Figure 3:**
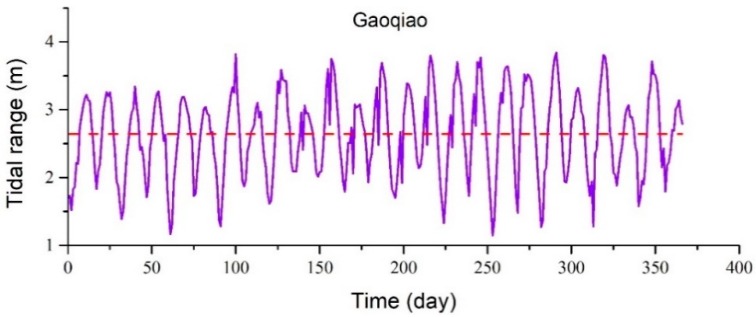
The tidal range with time in Gaoqiao station.

The variations in the daily tidal ranges over time at the tidal stations indicated that the tidal ranges had significant half-month periodic variations, along with changes during the year [30]. The tidal range of Gaoqiao Station was determined to be between 1 and 4 m (As shown in Figure 3).

#### 2.2.2. The Mechanism

In a general sense, the origin and development of any event are the accumulation of energy due to the growth of quantity. To a certain extent, it must be released. At the same time, in a general sense, the impact of resistance is always present during the development of an event. The accumulative process does not present a uniform linear growth law, but instead exhibits a nonlinear growth law under the influence of the impedance effect, and experiences three stages: initial, development, and end. The origin, development, and ending of the event are actually the result of the action of two opposing factors: one is the driving force that drives the accumulation and release of energy, and the other is the strength of resistance that hinders the development of the two events [31].

**Figure 4:**
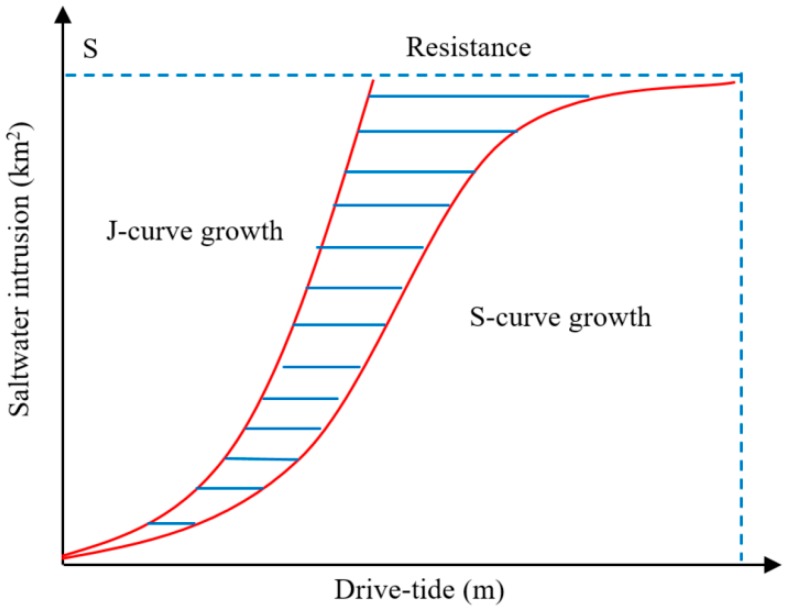
The salt water intrusion process curve.

When there is no resistance (an ideal environment), the saltwater intrusion intensity increases into a J-shaped curve with an increase in the grade of tide. However, this ideal situation is rarely found in nature. Generally, in an imperfect environment where environmental resistance is present (river discharge), the saltwater intrusion damage is offset by resistance to a certain extent. Therefore, the saltwater intrusion intensity can be characterized as an S-shaped curve (Figure 4) with a slow increase in the early stages, an accelerated increase in the intermediate stages, and stability in the final stages.

### 2.3. Function Curve Construction

#### 2.3.1. Function

**Figure 5:**
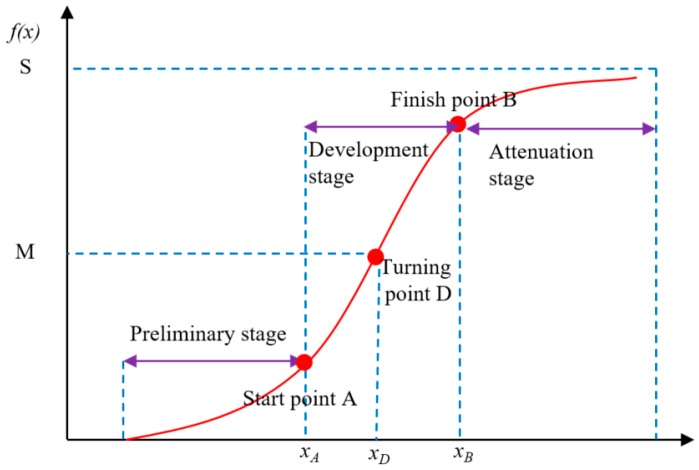
A conceptual model of the saltwater intrusion function.

The saltwater intrusion process was observed to conform to an “S” pattern of change, and was often subtle and sudden. Also, the mutation process was found to be obvious. It was determined to be similar to the loss change processes during water disasters (Figure 5) [31]. Therefore, the formula for constructing a saltwater intrusion process in this study was as shown in Formula (1).

(1) $$f\left(X\right)=\frac{S}{2}\left(\frac{e^{2\alpha \left(X-X_{D}\right)}-1}{e^{2\alpha \left(X-X_{D}\right)}+1}+1\right)$$

where *X* represents the driving or impedance factor during the saltwater intrusion process; S denotes the maximum limit value during the saltwater intrusion process (selected saltwater intrusion area); the parameter *α* is the vulnerability coefficient; and $X_{B}$ represents the finish point in the saltwater intrusion process. It was determined in the saltwater intrusion process function that the higher the vulnerability coefficient *α* was, the greater the salinity exceeded the standard area under the same conditions; and $X_{D}$ is the central symmetry point of the curve. Also, the same $X_{A}$ and $X_{B}$ denote the critical values of the salinity.

#### 2.3.2. Parametric Mathematics

In accordance with the function curve of the saltwater intrusion process shown in Figure 5 and Formula (1), the main parameters that affected the entire curve change process were the vulnerability coefficient α, the maximum saltwater intrusion area K, and the critical salinities $X_{A}$, $X_{D}$, and $X_{B}$.

Then, according to the nature of the curve, when $X=X_{D}$, the curve slope obtains the maximum value, or $k_{max}=\alpha S$. Therefore, the function slope had a correlation with the coefficient *α*, and the higher the system vulnerability was, the faster the loss change rate would be, and vice versa. At this point, from the symmetry of the S curve and the third derivative of the function at the inflection point, the critical salinities $S_{A},\;S_{B},\;\mathrm{and}\;S_{C}$ and the vulnerability coefficient α could be calculated as follows:

(2) $$X_{A}=X_{D}-\frac{0.66}{\alpha };\;X_{B}=X_{D}+\frac{0.66}{\alpha };$$

(3) $$X_{D}=\frac{X_{A}+X_{B}}{2};\;\alpha =\frac{1.32}{X_{B}-X_{A}}.$$

From the parameter calculation formulas, it can be seen that any two parameters that are known in the four parameters $S_{A},S_{B},S_{C},$ and α could be used to solve the other two parameters. However, it should be pointed out that, although $X_{D}$ can be theoretically solved by $X_{A}$ and $X_{B}$, the actual situation is more complicated. Therefore, this method only represents a simplified treatment of the practical problems.

### 2.4. Model Creation

MIKE21 was used to create the hydrological model. Details of MIKE21 and the model’s creation are given in Xu et al. [32].

### 2.5. Model Calibration and Validation

**Figure 6:**
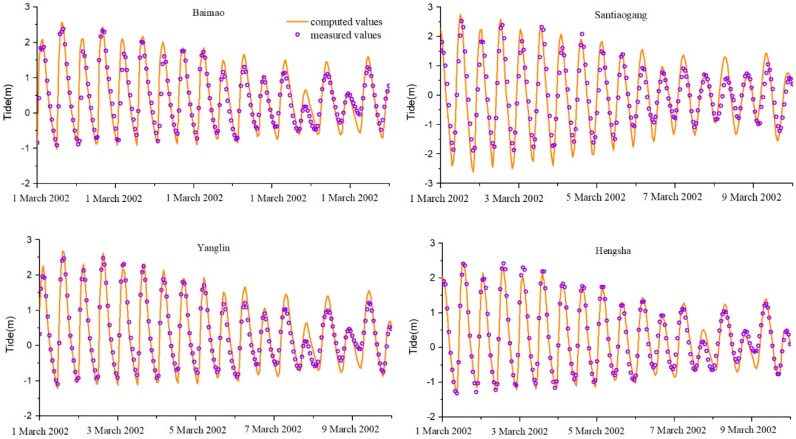
Verification of the tidal levels.

The hourly tidal level and salinity at eight stations were collected to verify the simulation (positions shown in Figure 1). The simulated water levels of the tidal stations (Baimao (Bm), Santiaogang (Stg), Yanglin (Yl), and Hengsha (Hs)) are in excellent agreement with the measured values (Figure 6).

**Figure 7:**
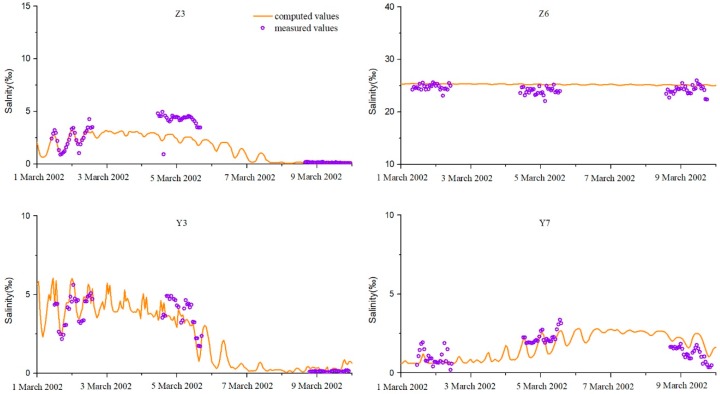
Verification of the salinity levels.

The salinity was measured as chlorination, and the Knudsen salinity formula [33] was used for the conversion. The verification results (Z3, Z6, Y3, and Y7) are shown in Figure 7.

The model was quantitatively assessed using the skill model [34]. The model is:

(4) $$skill=1-\frac{\sum_{i=1}^{N}{\left|M-X\right|}^{2}}{\sum_{i=1}^{N}{\left(|M-\overset{-}{X}|+|M-\overset{-}{X}|\right)}^{2}}$$

where *M* is the computed values; *X* is the measured values; $\overset{-}{X}$ is the mean value of *X*; and *N* is the number of in situ observed data. A *skill* value of 1 indicates that the simulation effect is exactly the same, a *skill* value between 0.65 and 1 means excellent, a *skill* value in the range of 0.5–0.65 means very good, a *skill* value in the range of 0.2–0.5 means good, and a *skill* value less than 0.2 means bad.

A time series of hourly tidal level and salinity at eight stations (positions shown in Figure 1) spanning the period from 1 March 2002 to 10 March 2002 was collected to validate the simulations. The simulation results at Bm, Stg, Yl, Hs, Z3, Z6, Y3, and Y7 are in excellent agreement with the observations (Figure 6 and Figure 7). The skill values are 0.93, 0.91, 0.94, 0.91, 0.70, 0.82, 0.78, and 0.77 at the eight stations (Table 1).

By verification, all stations’ *skill* values are greater than 0.65, which is excellent. Among them, four tidal station fitting values are above 0.9 (Bm, Stg, Yl, and Hs), and the salinity station *skill* values range from 0.70 to 0.82 (Table 1). This is because the salinity simulation depends largely on the flow rate change. The direction and intensity of seawater flow affect the diffusion of salinity [35]. Some of the upstream salinity stations have low salinity and are very sensitive to water flow. There will be some deviation during the simulation.

## 3. Results

### 3.1. Analysis of Salinity Distribution in the South Branch

Because the Yangtze River estuary reservoirs are distributed in the south branch, and the north branch salinity has been in the state of exceeding the standard for a long time, this paper mainly studies the change of salinity in the south branch.

#### 3.1.1. Salinity Changes in Different River Discharges

The river discharges of 10,000 m^3^/s, 15,000 m^3^/s, 20,000 m^3^/s, 25,000 m^3^/s, 30,000 m^3^/s, and 35,000 m^3^/s were modelled. We did not change the other conditions. The study focused on salinity changes at eight stations (S1, S2, S3, S4, S5, S6, S7, S8), which are shown in Figure 1.

**Figure 8:**
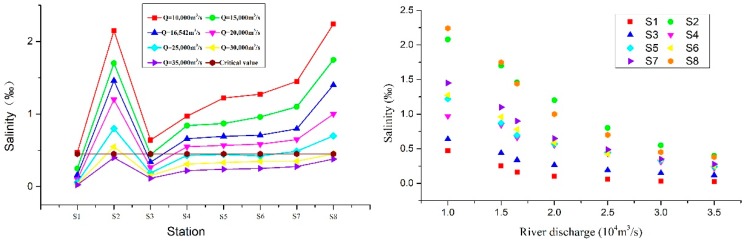
The average salinity values of the stations under different flow conditions.

Table 2 and Figure 8 show the average salinity values of the SB under different flow conditions. It can be seen that, with the decrease of the river discharge levels, the salinity distribution of the SB in the YRE displayed a “high-low-high” trend from the top to the bottom. The “high” point shown in the upper segment was located at the S2 point near the north-south branch junction; the “low” point was located in the middle segment S3–S5. As the salinity continued to rise, the “high” point in the lower segment was located near point S1 in the mouth of the river, and was mainly influenced by the offshore tidal flows.

Figure 8 shows that there was a negative correlation between the flow into the sea and the salinity levels measured at the stations. With increased but low flow rates, the salinity at each station decreased a little. In this study, prior to the flow rates in the sea reaching 10,000 m^3^/s, the salinity levels at each site were measured. It was found that, when the degree exceeded the standard, a full saltwater intrusion had occurred in the southern branch. Furthermore, when the flow rate into the sea was between 10,000 and 30,000 m^3^/s, the salinity values of the stations decreased rapidly. When the river discharge reached more than 30,000 m^3^/s, it was found that all of the measuring points (with the exception of point S2) were low. Therefore, it was confirmed that no salt water intrusions occurred when the salinity critical value was below 0.45.

#### 3.1.2. Salinity Changes in Different Tidal Ranges

The average annual dry season flow into the sea of Datong Station was confirmed to be 16,542 m^3^/s. A model of the flow boundaries was used to simulate six different tidal ranges (Table 3). The average salinity values of eight stations at different tidal ranges were then examined.

**Figure 9:**
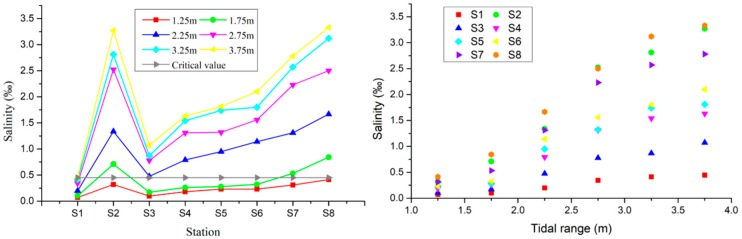
The average salinity values of stations at different tidal ranges.

Table 3 and Figure 9 show the average salinity distribution at the southern branch of the YRE under the different tidal ranges. Figure 1 shows the changes in average salinity value of different stations, along with changes in tidal ranges and salinity values at the different stations. As can be seen from the chart, the salinity value of stations also increased when the tidal ranges increased. It can also be seen in the figure that, along the southern branch (from top to bottom), the stations generally showed rising trends. This was due to the fact that the measuring point S2 was affected by the salt water intrusions of the northern branch, resulting in a higher salinity than the nearby stations. Furthermore, the salinity value of the SB1 station in the upper part of the southern branch was lower than 0.45‰ in all cases, and the salinity value of the S1 station was higher than the other stations.

Figure 9 shows that there is a positive correlation between tidal range and salinity at every station. With the increase of tidal range, the salinity of station slowly increases, and the salinity intrusion intensity is very weak before the tidal range reaches 1.75 m. At the tidal range of 1.75–3.5 m, the salinity at the stations increased rapidly, and the salinity invading rate accelerated. When the tidal range reached above 3.5 m, the rising rate of salinity slowed down and stabilized, and the stations continued to be at high salinity.

### 3.2. Analysis of Salinity Excessive Area Rate in the South Branch

To further quantitatively study the saltwater intrusion process, the typical river discharge (8000 m^3^/s, 10,000 m^3^/s, 15,000 m^3^/s, 20,000 m^3^/s, 25,000 m^3^/s, 30,000 m^3^/s, and 35,000 m^3^/s), tidal range (1.5 m, 2 m, 2.5 m, 3 m, and 3.5 m), and the relationship with the excessive Saltwater intrusion rate in the south branch were analyzed. The total area of the south branch of the YRE S (1901.86 km^2^). According to the model calculation, under the condition of a different runoff into the sea, and the excess area of the south branch $S_{s}$, the ratio of the exceeding area is:

(5) $$\varphi =\frac{S_{s}}{S}.$$

**Figure 10:**
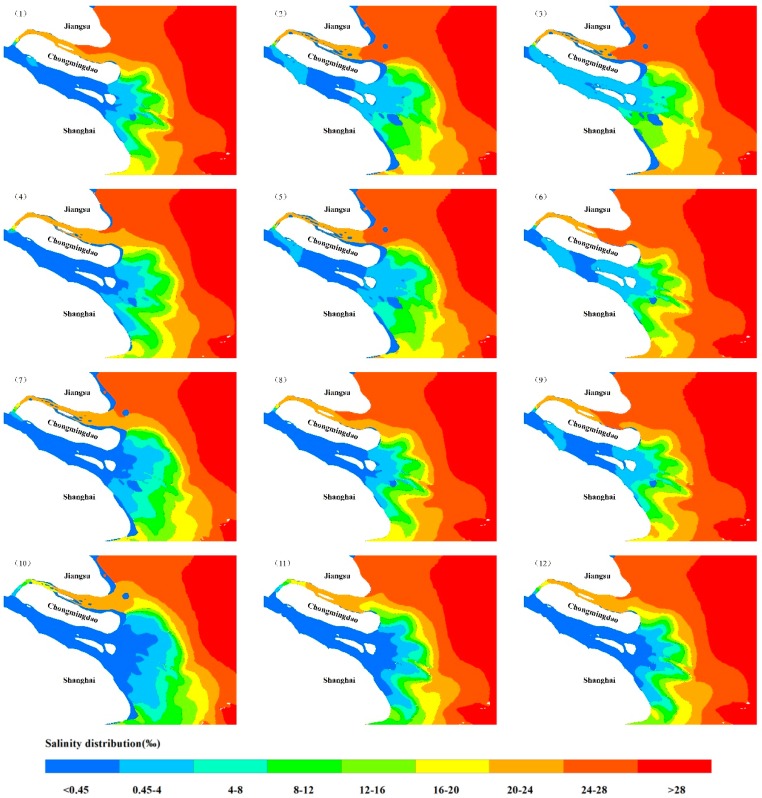
The salinity excessive area of the South Branch in different tidal range and river discharge conditions. Note: (1) Q = 10,000 m^3^/s, T = 1.5 m; (2) Q = 10,000 m^3^/s, T = 2.5 m; (3) Q = 10,000 m^3^/s, T = 3.5 m; (4) Q = 15,000 m^3^/s, T = 1.5 m; (5) Q = 15,000 m^3^/s, T = 2.5 m; (6) Q = 15,000 m^3^/s, T = 3.5 m; (7) Q = 20,000 m^3^/s, T = 1.5 m; (8) Q = 20,000 m^3^/s, T = 2.5 m; (9) Q = 20,000 m^3^/s, T = 3.5 m; (10) Q = 30,000 m^3^/s, T = 1.5 m; (11) Q = 30,000 m^3^/s, T = 2.5 m; (12) Q = 30,000 m^3^/s, T = 3.5 m.

As can be intuitively seen in Table 4 and Figure 10, the SEAR in the SB has increased when the tidal range increases under the same flow conditions. Moreover, at the same tidal range, with the increased flow levels, it was observed that the SEAR in the southern branch had also increased. When the flow rates into the sea were less than 10,000 m^3^/s, the saltwater began to completely invade the area. However, when the runoff from the sea reached 30,000 m^3^/s, basically no saltwater intrusion was observed.

**Figure 11:**
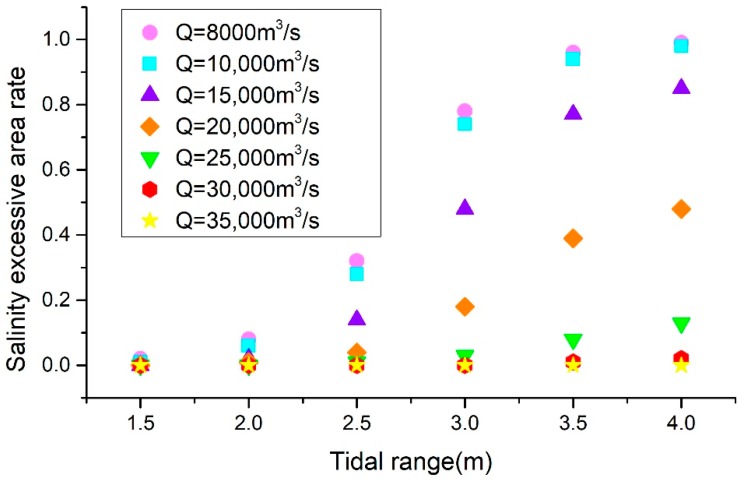
The salinity excessive area of the South Branch in different tidal range and river discharge conditions.

Figure 11 details the relationship between the tidal ranges and the SEAR at the different flow rates. It can be seen that the tidal ranges were positively correlated with the SEAR. Under the same flow conditions, the salinity exceedance rate was found to have increased with the increases in the tidal ranges. Also, at the same tidal range, the greater the flow rate into the sea, the less the SEAR of the southern branch. It was observed that when the tidal range was less than 1.75 m, the SEAR was lower than 0.1. However, as the tidal ranges increased, the SEAR rapidly increased when the tidal range was between 1.75 and 3.5 m. When the tidal ranges were higher than 3.5 m, the SEAR tended to become stable, and the salinity exceeding the standard area had reached its maximum level.

**Figure 12:**
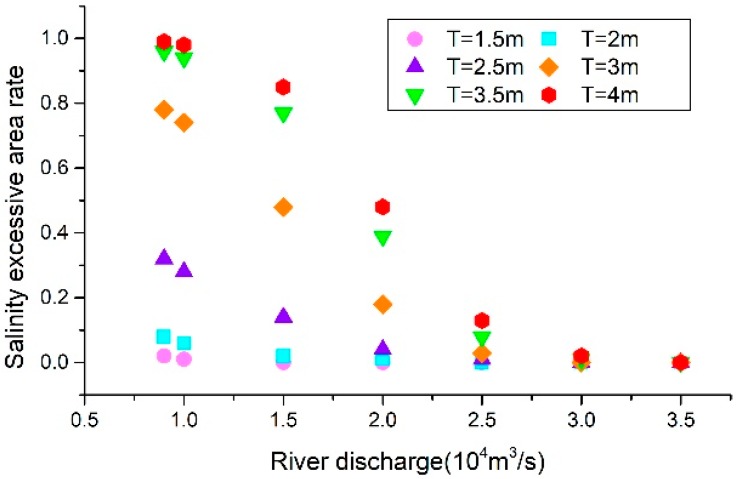
The relationship between the river discharge and the SEAR in different tidal range conditions.

Figure 12 shows the relationships between the tidal range under the different river discharge conditions and the SEAR in the southern branch. It can be seen in the figure that the flow into the sea was negatively correlated with the SEAR. Under the same tidal range conditions, as the flow increased, the SEAR increased. When the flow rate was less than 10,000 m^3^/s, the salinity exceedance rate was observed to become stable at the highest value. As the flow rate increased, it could be seen that the SEAR had rapidly dropped when the flow rate was between 10,000 and 30,000 m^3^/s. Then, when the flow rate into the sea was higher than 30,000 m^3^/s, the over-standard salinity tended to become stable, and the SEAR had reached the minimum.

### 3.3. Function Curve Determination

In this research study, through the coupling of the function change, quantum energy accumulation, and impedance action, the saltwater intrusion function in the YRE was constructed using both measured and simulated data from the YRE. Under the action of the flow series $x_{1},x_{2},\ldots ,x_{n}$ and the tidal range (*t*), the function between the saltwater intrusion with the tidal range (*t*) is shown in Equation (6). Also, the function of the maximum area of saltwater intrusion with the river discharge is shown in Equations (7) and (8).

(6) $$\alpha (x_{i})=f(t,x_{i})=\frac{A\left(x_{i}\right)}{2}\times \left(\frac{e^{2\alpha_{1}\times (t-t_{D})}-1}{e^{2\alpha_{1}\times (t-t_{D})}+1}+1\right)$$

(7) $$F(t,x_{i})=\frac{A_{\max}}{4}\times \left(\frac{e^{2\alpha_{1}\times (t-t_{D})}-1}{e^{2\alpha_{1}\times (t-t_{D})}+1}+1\right)\times \left(\frac{e^{2\alpha_{2}\times ({\hat{x}}_{iD}-{\hat{x}}_{i})}-1}{e^{2\alpha_{2}\times ({\hat{x}}_{iD}-{\hat{x}}_{i})}+1}+1\right)$$

(8) $$A_{\max}=\max(A\left(x_{i}\right))$$

In the equations, $x_{i}$ represents the river discharge; t is the tidal range; $A(x_{i})$ is the maximum area of the salinity intrusion under the $x_{i}$ condition; $\alpha_{1}$ is the sensitivity coefficient of coverage area to the tidal range during the salt tide intrusion; $t_{D}$ is the central symmetry point of the curve; $\alpha_{2}$ denotes the sensitivity coefficient of the maximum intrusion area’s influencing factors during the salt tide intrusion response process; ${\hat{x}}_{i}$ represents the dimensionless value of the inflow into the sea (including the measured river discharge and the average annual river discharge); and ${\hat{x}}_{iD}$ is a function of the dimensionless sea inflow and the salt tide intrusion response function curve’s turning point.

#### 3.3.1. Function Determination for the SEAR and Tidal Ranges

**Figure 13:**
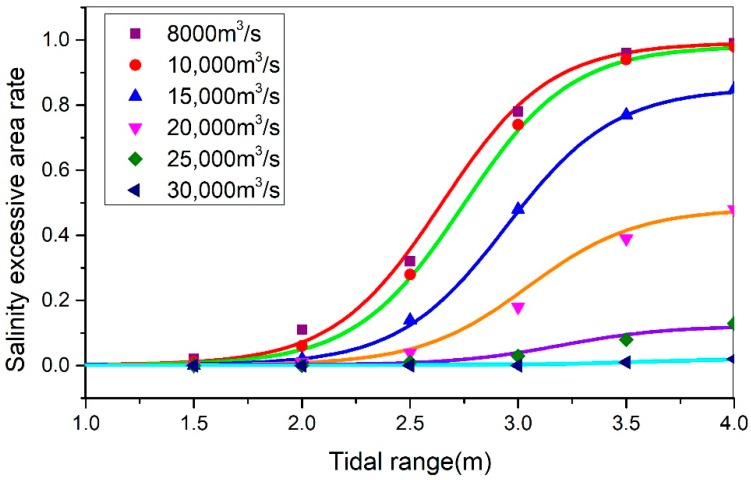
The function curve between the tidal range and the SEAR in different river discharge conditions.

In this study, by using multiple parameter calibrations and curve fittings, the sensitivity coefficient $\alpha_{1}$ = 2 was successfully determined, and the relevant parameters are shown in Table 5. The curve of the function of the SEAR with the change of the tidal ranges under the different river discharge conditions is shown in Figure 13.

It can be seen from Figure 13 that, as a group, the curve of the saltwater intrusion area with the changes in the tidal ranges was within the same curve family. Its parameters had also followed certain rules. According to Table 5, the relationship between the transition tidal range $t_{D}$ and river discharge is as follows: when the river discharge increases, $t_{D}$ increases. Indirectly, the larger the discharge is, the greater the tide that causes saltwater intrusion is. The side shows that river discharge acts as an impedance in the saltwater intrusion process.

#### 3.3.2. Function Determination for the SEAR and River Discharge Conditions

The accurate determination of the relationship between the runoff from the sea and the maximum area of the saltwater intrusion was the precondition for determining the boundary parameter $A\left(x_{i}\right)$ in the saltwater intrusion response function. In this study, through the use of multiple parameters, the parameters were obtained as follows: ${\hat{x}}_{iD}$ = 0.72, $\alpha_{2}$ = 5.0, and $A_{\max}$ = 1. Therefore, the function was as follows:

(9) $$A(x_{i})=0.5\times \left(\frac{e^{10.0\times (0.72-{\hat{x}}_{i})}-1}{e^{10.0\times (0.72-{\hat{x}}_{i})}+1}+1\right).$$

**Figure 14:**
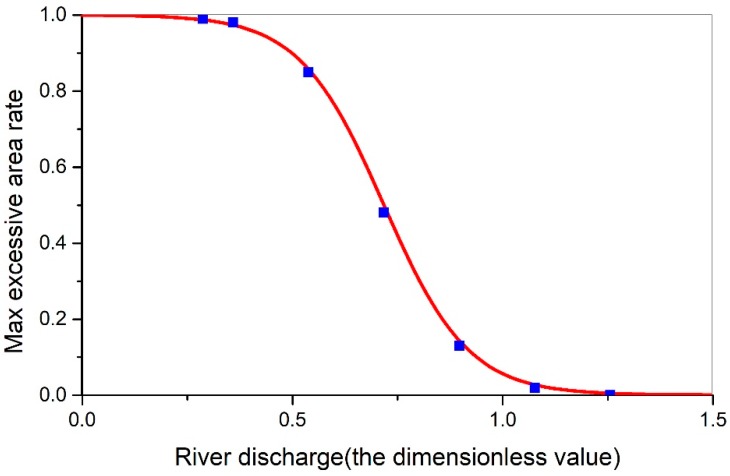
The function curve between river discharge and maximum excessive area rate.

As can be seen from Figure 14, the function fitting effect is good, and there is an S-type variation pattern between the maximum rate of salinity exceeding the standard and the flow rate into the sea.

#### 3.3.3. Function of the Saltwater Intrusion Process in the YRE

In this study, the relationships between the intrusion areas with the salt tide and the changes in the tidal ranges, and the relationships between the maximum area of the salt tide intrusion and the flow rate into the sea, were used to obtain the theoretical function of the saltwater intrusion, along with the diffusion response process in the estuary, as shown in Equation (8). From the calculation and solution of the measured data detailed in the previous two sections, the parameters that could be determined in the theoretical function were as follows: ${\hat{x}}_{iD}$ = 0.72, $\alpha_{1}$ = 2.0, $\alpha_{2}$ = 5.0, and $A_{\max}$ = 1. Therefore, the saltwater intrusion diffusion response function in the YRE could be further transformed into Equation (10).

(10) $$\begin{array}{l} F(t,x_{i})=\frac{A_{\max}}{4}\times \left(\frac{e^{2\alpha_{1}\times (t-t_{D})}-1}{e^{2\alpha_{1}\times (t-t_{D})}+1}+1\right)\times \left(\frac{e^{2\alpha_{2}\times ({\hat{x}}_{iD}-{\hat{x}}_{i})}-1}{e^{2\alpha_{2}\times ({\hat{x}}_{iD}-{\hat{x}}_{i})}+1}+1\right)\\ \;=0.25\times \left(\frac{e^{4\times (t-t_{D})}-1}{e^{4\times (t-t_{D})}+1}+1\right)\times \left(\frac{e^{10.0\times (0.72-{\hat{x}}_{i})}-1}{e^{10.0\times (0.72-{\hat{x}}_{i})}+1}+1\right) \end{array}$$

**Figure 15:**
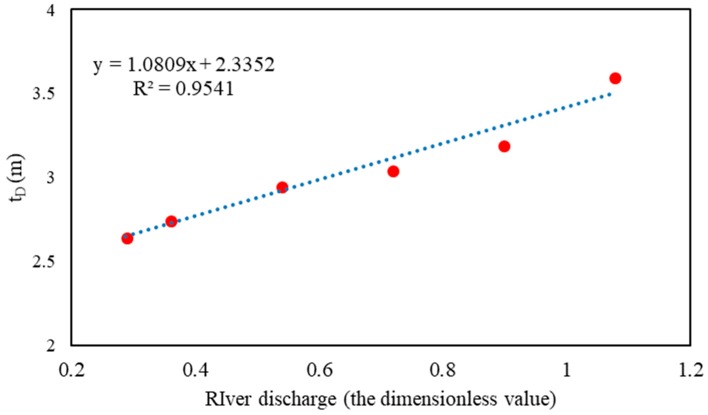
The relationship between dimensionless river discharge and transition tidal range ($t_{D}$).

Among them, $t_{D}$ changes with the change of runoff into the sea, and, according to the relevant parameter values determined in Table 5, the relationship between the dimensionless flow into the sea and the $t_{D}$ can be obtained (see Figure 15). The relationship between the two is brought into Equation (10), and Formula (11) is obtained as follows:

(11) $$F(t,x_{i})=0.25\times \left(\frac{e^{4\times \left(t-(1.0809{\hat{x}}_{i}+2.3352)\right)}-1}{e^{4\times \left(t-(1.0809{\hat{x}}_{i}+2.3352)\right)}+1}+1\right)\times \left(\frac{e^{10.0\times (0.72-{\hat{x}}_{i})}-1}{e^{10.0\times (0.72-{\hat{x}}_{i})}+1}+1\right).$$

**Figure 16:**
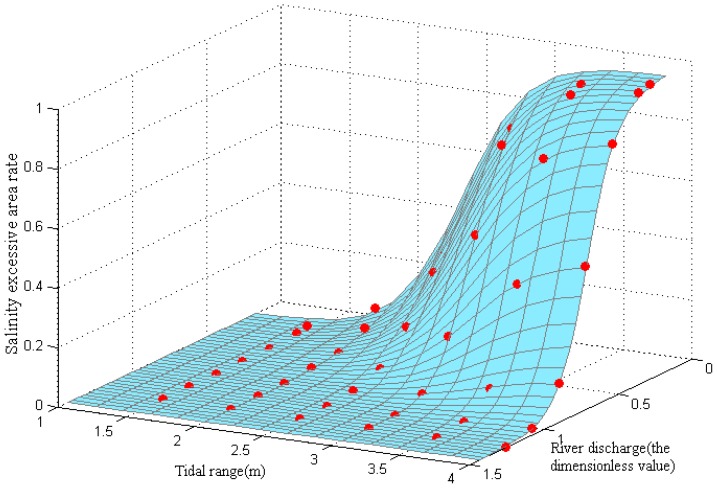
The scatter diagram and saltwater intrusion function surface fitting.

Figure 16 details the simulated scatter distribution and function fitting curved surface. It can be seen in the figure that the tidal ranges played key roles in promoting the SEAR, and the river discharge conditions played key roles in resisting the SEAR. The former (tidal ranges) were the driving forces of the saltwater intrusions. The latter (river discharge conditions) were the group resistance to the saltwater intrusions. It was determined that, under any of the flow conditions into the sea, as the tidal range increased, the SEAR consistently changed from a slow to a rapid expansion, and then tended to ease once it approached the maximum area. The three factors (tidal range, river discharge, and SEAR) were found to be related to each other and had formed an S-shaped curved surface that conformed to the principles of the interactions between the amount of accumulated energy and the impedance. The SEAR was observed to have gradually increased with the tidal range above approximately 1.75 m (in which the runoff was less than 10,000 m^3^/s). Also, after entering the sea with a runoff greater than 30,000 m^3^/s, it was observed to be difficult for the tidal ranges that exceeded 3.75 m to form an excess area.

### 3.4. Saltwater Intrusion Damage Assessment

According to some studies in recent years, the value of ecosystem services per unit area in the YRE is between 117.91 and 189.44 million RMB/m^2^ [25,26]. In this paper, the average value of 1.5 million RMB/km^2^ is taken for analyzing the loss. Using Formula (9), the maximum intrusion area of the SB in the YRE is calculated by the minimum sea runoff from November to April of the 2005–2015 dry season (from November to April of the following year), and the maximum loss of saltwater intrusion in the YRE is calculated. Since the NB accounts for about 24% of the YRE’s area, and the salinity of the NB’s gate is equivalent to that of the outer sea during the dry season, the salinity of the upper section of Qinglong Port is also higher than the set salinity standard value, so the salt intrusion area of the entire Yangtze River estuary is south. The sum of the intrusion area of the saltwater and the area of the NB is used to evaluate the saltwater intrusion loss in the YRE. The maximum loss in the dry season from 2005 to 2015 is shown in Table 6.

## 4. Discussion

This research studied the numerical simulation results of saltwater intrusion in the YRE, and studied the saltwater intrusion and diffusion response from the hydrological factors that dominate the saltwater intrusion. In future research, many problems should be explored:

The study of the saltwater intrusion and diffusion process considers the main hydrological factors in the process of saltwater intrusion: river discharges and tides. However, the environment in the estuary area is complex. Under the influence of natural and human factors, the river regime changes, and the estuary’s water conservation construction project, the change of wind stress may have a certain impact on the original saltwater intrusion process, which affects the accuracy of the diffusion response function. The above influencing factors should be gradually introduced in the future. On the basis of studying the influence of the above factors on the salt tide intrusion process, the parameters reflecting the above factors should be introduced to correct the saltwater intrusion diffusion response function.

The paper takes the YRE as an example for empirical analysis and function construction that is based on the hydrological response mechanism of saltwater intrusion. In China, in addition to the YRE, areas such as the Pearl River estuary and the Qiantang estuary are also facing serious saltwater intrusion, as well as in some estuaries of other countries. In future research, we will further expand the empirical analysis into other estuary areas. The applicability of the saltwater intrusion function in other regions should be further studied.

The calculation results of the numerical simulation can be used as the basic data of the saltwater intrusion process to ensure the richness of the research data. In order to improve the accuracy of the saltwater intrusion analysis, it is also necessary to enrich the hydrological data in the estuary area and increase the accuracy of the numerical simulation in order to more accurately evaluate the saltwater intrusion and diffusion process.

## 5. Conclusions

In this study, based on previous research results regarding the quantum energy accumulation and impedance action theory, the saltwater intrusion processes that occur in the YRE under the interactions of the tide rates and runoff into the sea were examined. It was concluded from the research findings that the tidal water rates were the driving forces for the saltwater intrusions. The impedance of the intrusions was determined to be consistent with the theory. Also, an S-curve function, which conformed to the theory of energy accumulation and impedance, was successfully constructed. The parameter settings and quantification methods were described in detail in the current study. A MIKE21 model was used to simulate the hydrodynamic processes and salinity diffusion in the YRE. First, a linear analysis was performed on eight stations that covered the southern branch of the YRE. The calculation results of the salinity levels from the upstream to the downstream of the southern branch of the YRE were presented. The characteristic of a “high-low-high” change trend was determined to be due to the influences of the southern branch’s high brine concentration offshore, as well as the salinity at the SB2 station near the northern branch being significantly increased. These findings were found to be in line with the research results of previous related studies. A linear analysis of the salinity exceeding standard area (SEAR) of the southern branch was carried out. The exceeding rates of the flow and salinity, and the exceeding rate of the tidal differences and salinity, were found to be in line with the change rule of the previously mentioned S curve.

Also, through the determination of the brine process function, two critical sea inlet flows were determined in this study as follows: When the seawater flows were greater than 10,000 m^3^/s, which could withstand large-scale saltwater intrusions; and when the seawater flows were greater than 30,000 m^3^/s, where no saltwater was observed to intrude, and the estuarine ecology was in an optimal state. The saltwater intrusion losses from 2005 to 2015 were also calculated. The results of this study will potentially provide important reference values for future water scheduling during the dry seasons in the upper and middle reaches of the YRE. This important information will further the comprehensive management of the estuary in the future.

## Figures and Tables

**Table 1:** Model–observation data comparison statistics for tidal and salinity levels.

Quantity	Stations	*Skill*	Condition
Tidal current	Bm	0.93	Excellent
	Stg	0.91	Excellent
	Yl	0.94	Excellent
	Hs	0.91	Excellent
Salinity	Z3	0.70	Excellent
	Z6	0.82	Excellent
	Y3	0.78	Excellent
	Y7	0.77	Excellent

**Table 2:** The average salinity values of the southern branch (SB) under different flow conditions.

Station	Average Salinity Values with the Discharge of 16,542 m^3^/s (‰)	Average Salinity Values with Different Flow Conditions.					
		10,000 m^3^/s (‰)	15,000 m^3^/s (‰)	20,000 m^3^/s (‰)	25,000 m^3^/s (‰)	30,000 m^3^/s (‰)	35,000 m^3^/s (‰)
S1	0.05	0.10	0.05	0.04	0.03	0.03	0.02
S2	1.46	2.28	1.70	1.20	0.80	0.55	0.40
S3	0.34	0.76	0.41	0.26	0.19	0.15	0.12
S4	0.73	1.05	0.83	0.61	0.47	0.36	0.26
S5	0.69	1.22	0.80	0.57	0.44	0.33	0.24
S6	0.71	1.27	0.82	0.58	0.42	0.35	0.25
S7	0.80	1.67	0.94	0.65	0.49	0.35	0.28
S8	1.50	2.97	1.75	1.00	0.70	0.45	0.38

**Table 3:** The average salinity values of SB at different tidal ranges.

Station	Average Salinity Value at Different Tidal Range (‰)					
	<1.5 m (‰)	1.5–2 m (‰)	2–2.5 m (‰)	2.5–3 m (‰)	3–3.5 m (‰)	>3.5 m (‰)
S1	0.070	0.101	0.198	0.343	0.412	0.445
S2	0.320	0.710	1.337	2.524	2.810	3.270
S3	0.098	0.170	0.475	0.778	0.868	1.072
S4	0.181	0.261	0.791	1.310	1.540	1.630
S5	0.230	0.279	0.949	1.320	1.740	1.810
S6	0.231	0.321	1.140	1.560	1.800	2.100
S7	0.310	0.532	1.310	2.230	2.570	2.780
S8	0.412	0.843	1.666	2.500	3.120	3.330

**Table 4:** The South Branch’s salinity excessive area rate (SEAR) in different river discharge and tidal range conditions.

	Tidal Range (m)	1.5	2	2.5	3	3.5	4
River Discharge (m^3^/s)							
8000		0.02	0.11	0.32	0.78	0.96	0.99
10,000		0.01	0.06	0.28	0.74	0.94	0.98
15,000		0	0.02	0.14	0.48	0.77	0.85
20,000		0	0.01	0.04	0.18	0.39	0.48
25,000		0	0	0.01	0.03	0.08	0.13
30,000		0	0	0	0	0.01	0.02
35,000		0	0	0	0	0	0

**Table 5:** The saltwater intrusion area and tidal changes relationship parameters.

***Q* (m^3^/s)**	8000	10,000	15,000	20,000	25,000	30,000	35,000
$A_{i\max}$ **(%)**	0.99	0.98	0.85	0.48	0.13	0.02	0
$t_{D}$ **(m)**	2.65	2.75	2.95	3.05	3.2	3.6	-

**Table 6:** The maximum loss by saltwater intrusion in the Yangtze River Estuary (YRE).

Year	Minimum River Discharge (m^3^/s)	Maximum Excess Area (km^2^)	Maximum Loss (100 Million RMB)
2005	11,600	2062.40	15.84
2006	9650	2130.87	16.37
2007	10,000	2122.29	16.30
2008	10,300	2113.85	16.23
2009	10,900	2093.44	16.08
2010	11,200	2081.19	15.98
2011	13,800	1892.00	14.53
2012	11,200	2081.19	15.98
2013	12,265	2021.43	15.52
2014	11,271	2076.56	15.94
2015	12,377	2014.73	15.47
